# Variability of a predator-prey interaction in the plankton: Encounters and feeding rates of the chaetognath *Flaccisagitta enflata* upon copepods

**DOI:** 10.1371/journal.pone.0340065

**Published:** 2026-01-02

**Authors:** Laura Sanvicente-Añorve, Elia Lemus-Santana, Gerardo Molina-Sandoval, Juan J. Cruz-Motta, Marco Violante-Huerta, Margarita Hermoso-Salazar

**Affiliations:** 1 Laboratorio de Ecología de Sistemas Pelágicos, Instituto de Ciencias del Mar y Limnología, Universidad Nacional Autónoma de México, Mexico City, Mexico; 2 Department of Marine Sciences, University of Puerto Rico, Mayaguez, Puerto Rico; 3 Facultad de Ciencias, Universidad Nacional Autónoma de México, Mexico City, Mexico; Central University of South Bihar, INDIA

## Abstract

This study examined the predator-prey interaction between *Flaccisagitta enflata*, a dominant chaetognath species, and copepods in the southwestern Gulf of Mexico to investigate the roles of environmental variables and predator-prey encounters in the feeding rate of *F. enflata* on copepods and to analyze the gut content of the predator throughout three seasons. Zooplankton samples were collected in summer, fall, and winter in both neritic and oceanic waters. Predator-prey encounters were examined under calm and turbulent conditions to test the influence of wind-induced turbulence. Results indicated that encounters varied across seasons and zones: under calm conditions, they ranged from 11 to 75 copepods/chaetognath.day; under turbulent conditions, encounters increased by 1.5 to 1.8 times at the surface. Statistical tests revealed significant differences in feeding rates across seasons and zones: in summer and fall, feeding rates were higher in neritic waters, whereas in winter, they were higher in the oceanic zone. The primary factors influencing feeding rates were temperature, encounter rates, and salinity. Higher summer and fall temperatures resulted in shorter digestion times and, consequently, higher feeding rates (0.44 to 0.74 copepods/chaetognath day). The encounter rates, strongly correlated with copepod density, positively influenced feeding rates, particularly in summer and fall, with the highest values in the neritic zone. The lowest salinity records, caused by river discharges and observed in winter in shelf waters, corresponded with the lowest feeding rates (0.30 copepods/chaetognath.day). Freshwater inflows carrying suspended sediments increase turbidity, which potentially interferes with the predatory mechanisms of *F. enflata* and diminishes its feeding rates. Specifically, the main ingested copepods were members of the genera *Temora* and *Euaugaptilus.* These findings improve our understanding of the predator-prey interaction between the most abundant zooplankton organisms.

## Introduction

In aquatic environments, zooplankton organisms are typically scattered in space, but they often aggregate into dense clusters that differ in spatial extent [1,2]. Zooplankters are considered sentinels of the ocean’s changes due to their sensitivity to physical conditions, such as temperature, light, pressure, turbulence, and discontinuity layers, but also to ecological relationships, such as predation-prey interactions [2,3].

The concentration of organic matter suspended in the oceans varies between 10^−2^ and 10^−5^ mg C/cm^3^, equivalent to only a few grains of rice in a cubic meter of water [4]. Therefore, for small plankters, the ocean represents a highly diluted environment in which they must meet their nutritional and growth requirements [4,5]. Finding food, locating a mate, and avoiding predation are the three primary tasks that zooplankton must accomplish to survive in the pelagic environment, where encounters between individuals serve as the “currency” of biological interactions [6,7]. The success of an encounter depends on how densely packed the organisms are, how quickly the individuals move towards each other, and how far a searcher (i.e., predator) can detect a target (i.e., prey) [7]. Besides improving the chance of finding food or mates, the aggregation of zooplankton organisms may reduce predation risk at both individual and collective levels. In aggregations, swarms result from the clustering behavior of a single species, and patches occur when multiple species aggregate [2,8].

The swimming patterns of planktonic organisms are highly correlated with their feeding strategy [6]. Predators in the plankton exhibit two main feeding modes: the ambush and the cruising strategies. Ambush feeders are mostly stationary and attack specific prey within their perception sphere, eventually moving to test a new location. In contrast, cruising feeders swim actively through the water to locate and capture their prey [4,6,9]. A cruising predator has a higher probability of encountering prey or a mate, but also has a major risk of being eaten; thus, a trade-off between swimming capabilities and feeding strategies must exist to maximize the success of populations in nature [4]. In addition to swimming behavior, small-scale turbulence has a relevant impact on planktonic interactions [10–12]. In the oceans, the effect of wind-induced turbulence is most pronounced in the upper oceanic layer, where plankton is more abundant [12]. The effects of small-scale turbulence can be either beneficial or inimical for plankton trophodynamics [13,14]. However, these consequences depend not only on turbulence intensity but also on the particular features of planktonic organisms [14–17]. Thus, higher velocities of small-scale turbulence enhance the encounter rates between predator and prey, but at the same time, may decrease the captures of prey, making them more difficult to catch [18,19]; then, the optimal feeding strategy for a predator involves a trade-off between maximizing encounters with prey and minimizing difficulties for their capture [20].

The major component of the mesozooplankton, in terms of numerical abundance and biomass, corresponds to the copepods. These small crustaceans exhibit three main features that contribute to their dominance in the pelagic realm: their ability to detect and escape from predators, the capability of remotely perceiving their prey, and their efficiency in finding a mate in a highly diluted medium [21]. Crustacean copepods comprise between 55 and 95% in counts and up to 90–97% in biomass, depending on localities and seasons; so, their role in the transfer of energy from primary producers to higher trophic levels is undeniable [22]. Copepods are the major grazers of phytoplankton, form part of the microbial loops, consume a variety of zooplankton organisms, including small fish larvae, and serve as prey for fish larvae, chaetognaths, and other pelagic carnivores [23–26]. These planktonic predators may interact in a complex trophic dynamic. Chaetognaths and fish larvae, for instance, can be involved in an intraguild relationship, in which they may act as predators, competitors, or prey. Chaetognaths can prey on newly spawned larvae and have a significant impact on the fish population through direct predation and by competing for food items, such as copepods. In turn, when fish larvae grow, they could become chaetognath predators [23].

Chaetognaths are often the second most abundant group within the zooplankton, second only to copepods [27]. These animals are among the major predators in plankton communities, primarily feeding on copepods, often resulting in significant predation pressure on these small crustaceans [22,28,29]. The ambush-predatory behavior of chaetognaths is associated with their highly hydrodynamic body shape [4]. They can perceive the micro-hydrodynamical disturbance caused by their prey in the surrounding environment through sensory arrays of setae that cover their body [30]. Once detected, the prey can probably be captured with the grasping spines around the chaetognaths’ head, and then, they bite and introduce toxins to the prey with their teeth [22]. Among chaetognaths, *Flaccisagitta enflata* is frequently cited as a dominant species in several locations [31–35]. This species is widely distributed in the epipelagic layer of tropical and subtropical zones worldwide [36,37]. Because of its abundance, *F. enflata* has a high relevance in the trophic structure of planktonic food webs [28,38,39]; however, a deeper understanding of its ecological role requires addressing some critical questions: How does the micro-turbulence affect the encounters of chaetognaths and copepods? What factors primarily affect the feeding rates of chaetognaths? Do the encounters between predators and prey directly influence the feeding rates? Specifically, what are the main prey? This study examined the trophic interaction between the chaetognath *F. enflata* and the copepods in neritic and oceanic waters of the southwestern Gulf of Mexico. Notably, 1) we analyzed the seasonal variations in the predator-prey encounter rates and the feeding of *F. enflata* upon copepods, under calm and turbulent conditions, 2) we attempted to identify the main environmental variables influencing the feeding rates, and 3) we analyzed the gut contents of *F. enflata* and determined the main prey. The findings of this study will enhance the understanding of the trophic relationship between two major zooplankton groups, the chaetognaths and the copepods.

## Materials and methods

### The sampling

**Fig 1:**
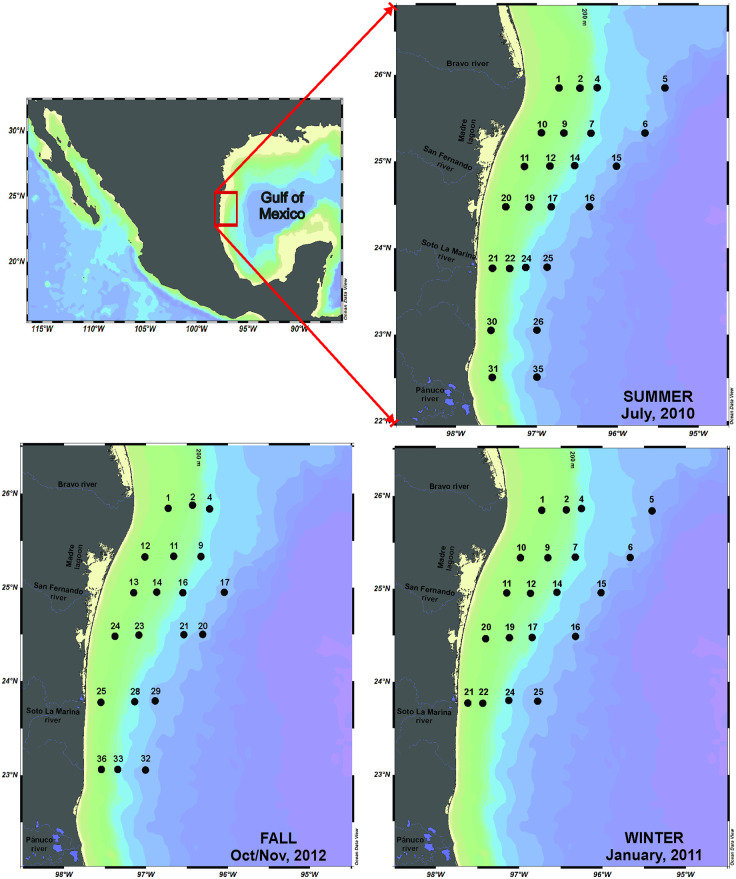
Location of zooplankton sampling stations in the southwestern Gulf of Mexico during three seasons. Republished from Ocean Data View under a CC BY license, with permission from Prof. Reiner Schlitzer (rights holder), original copyright 2025.

Zooplankton samples were obtained onboard the oceanographic vessel “Justo Sierra” in the southwestern Gulf of Mexico over a station grid that covered neritic and oceanic waters (Fig 1). Sampling was conducted throughout three seasons: summer (July 2010), fall (October-November 2012), and winter (January 2011). A total of 64 samples were collected using a Bongo net with mesh sizes of 333 and 505 micrometers; at each net mouth, a flowmeter was installed to estimate the volume of filtered water. Zooplankton collections were performed following oblique tows; the sampling depth varied between 10 and 200 m, and the towing time between 4 and 45 min, depending on the bathymetry. The samples were preserved with 4% formaldehyde buffered with sodium borate. Vertical profiles of temperature and salinity were obtained using a CTD profiler, and chlorophyll concentrations were also recorded with a sensor attached to the CTD sonde. For multivariate statistical analyses, we took the mean integrated value of environmental variables in the 50 m upper layer. Figures were created with Surfer and Ocean Data View version 5.8.3 [40] softwares.

### Laboratory and data analyses

From each sample, the predator *F. enflata* and the prey, the copepods, were separated, counted, and standardized to one cubic meter of water (ind/m^3^). We also separated up to 1000 individuals of the species *F. enflata*, which were identified according to specialized literature [41,42]. The digestive tract of each animal was carefully examined to count the number of ingested prey (copepods), except for those found near the mouth [43]; these data were used to calculate the mean number of copepods ingested by a chaetognath (*NPC*). Then, the feeding rate (*FR*) (number of copepods daily ingested by a chaetognath, copepods/chaetognath.day) was estimated according to $FR=\;\frac{NPC}{DT}(\text{2}\text{4})$ [44], where the *DT* is the digestion time in hours, calculated as $DT=\text{1}\text{0}.\text{4}\text{8}e^{-\text{0}.\text{8}\text{6}T}$, where *T* is the temperature [45], here integrated in the upper 50 m layer.

Encounter rates between the chaetognath predator *F. enflata* and its copepod prey were estimated for calm and turbulent conditions. For this purpose, we used two ecological models, the Gerritsen and Strickler model (GS) for calm condition simulations [46], and the Rothschild and Osborn model (RO) for turbulent conditions [18]. The GS model is defined by $C_{GS}=\frac{\pi R^{\text{2}}N}{\text{6}}\frac{(x+y{)}^{\text{3}}|x-y{|}^{\text{3}}}{xy}$, where *C*_*GS*_ is the number of prey encountered by a predator in a day (copepods/chaetognath.day), *R* is radius of encounter of the predator (m), *N* is the number of prey per m^3^, *x* is the prey velocity (m/s), and *y* is the predator velocity (m/s). The RO model adapted the GS model to introduce the effect of the small-scale turbulent velocity (*w*) induced by the wind when it blows over the ocean surface. Thus, for the encounter rates in the RO model (*C*_*RO*_), the *x* is replaced by $\sqrt{x^{\text{2}}+\;w^{\text{2}}}$, and the *y* by $\sqrt{y^{\text{2}}+\;w^{\text{2}}}$. The turbulent velocity (*w*) corresponds to the root-mean-square of the turbulent kinetic energy (*k*). The parameters *w* and *k* were estimated as previously exposed [10,11], using wind velocity data of the Windy Weather Service platform [47].

The swimming speed of the predator *F. enflata* was assumed to be zero, due to its ambushing predatory behavior, and the *R*, its prey detection radius, was set at 3 mm [48], in agreement with previous observations. For copepods, the prey, the escape response speed was 150 mm/s [49]. Mean values of wind velocities were 2.64 m/s for July, 2.81 m/s for October, and 3.87 m/s for January [47].

To detect differences in feeding rates among seasons (summer, fall, winter) and zones (neritic, oceanic), we performed a PERMANOVA test [50] with seasons and zones as factors. Before its application, data on feeding rates were subjected to a Euclidean distance to construct a similarity matrix. Additionally, a BEST BIOENV [51] test was applied to determine the explanatory variables (temperature, salinity, chlorophyll, bottom depth, distance from the coast, predator/prey encounter rates with RO model) that best explain the variability in the response variable, the feeding rates of chaetognaths on copepods. The copepod density was excluded from this analysis because of its high correlation with the encounter rates (as stated in the formulations above, encounter rates are directly related to copepod density and have *R*^*2*^ = 1). These multivariate techniques were performed with the PRIMER v7 with PERMANOVA + add-on [51] software. Data on encounters and feeding rates are available at https://doi.org/10.5281/zenodo.17517186.

Finally, for each sampling season, we took aliquots of predators containing copepods in their guts to measure the total length of both chaetognaths and copepods to the nearest 0.1 mm under a stereomicroscope. Then, chaetognaths were dissected to analyze their gut content and, when possible, the ingested copepods were identified to genus or species level. Prey items were classed as identifiable copepod, non-identifiable copepod, non-copepod prey, and non-identifiable prey. For the environment, we also took sample aliquots to identify copepods as specifically as possible using specialized literature [52,53]. Afterward, we applied the Kimmerer and Slaughter electivity index [54] to compare the feeding habits of *F. enflata* with the availability of food items in the environment. This index is defined by $X_{i}=\;\frac{O_{i}}{\text{1}+O_{i}}$, where *X_i_* is the electivity or degree of selection of the predator for a particular prey, and $O_{i}=\frac{\text{odds}G}{\text{odds}A}$, where $\text{odds}G=\frac{G_{i}}{N_{G}-G_{i}}=\frac{g_{i}}{\text{1}-g_{i}}$ and $\text{odds}A=\;\frac{A_{i}}{N_{A}-A_{i}}=\frac{a_{i}}{\text{1}-a_{i}}$. In these equations, *G*_*i*_ is the number of prey *i* in the guts, *N*_*G*_ the total of prey items in the guts, *A*_*i*_ is the availability of prey *i* in the environment, and *N*_*A*_, the total number of prey in the environment. Thus, $g_{i}=\frac{G_{i}}{N_{G}}$ and $a_{i}=\frac{A_{i}}{N_{A}}$ represent the proportions of prey item *i* in the gut of the predator and in the environment, respectively. The electivity index ranges from 0 to 1, where 0.5 indicates no selection, values above 0.5 indicate active selection, and values below 0.5 indicate prey avoidance. To estimate the index’s uncertainty, or confidence interval, a Beta-Monte Carlo simulation was implemented in R using the *rbeta* function. The process consists of generating 10,000 random samples (simulations) from beta-binomial distributions for *A*_*i*_ and *G*_*i*_ to compute *O*_*i*_ and, then, a new *X*_*i*_. Therefore, a 95% confidence interval was estimated to validate the observed electivity index value: if the interval includes 0.5, selection is not significant; if the interval is entirely above 0.5, it means positive selection; and if the interval is entirely below 0.5, it means negative selection. Finally, the dependence between the sizes of the predator and the prey was tested using a linear regression, in which we assessed whether the slope was significantly different from zero with a *t*-test.

## Results

### The environment

**Fig 2:**
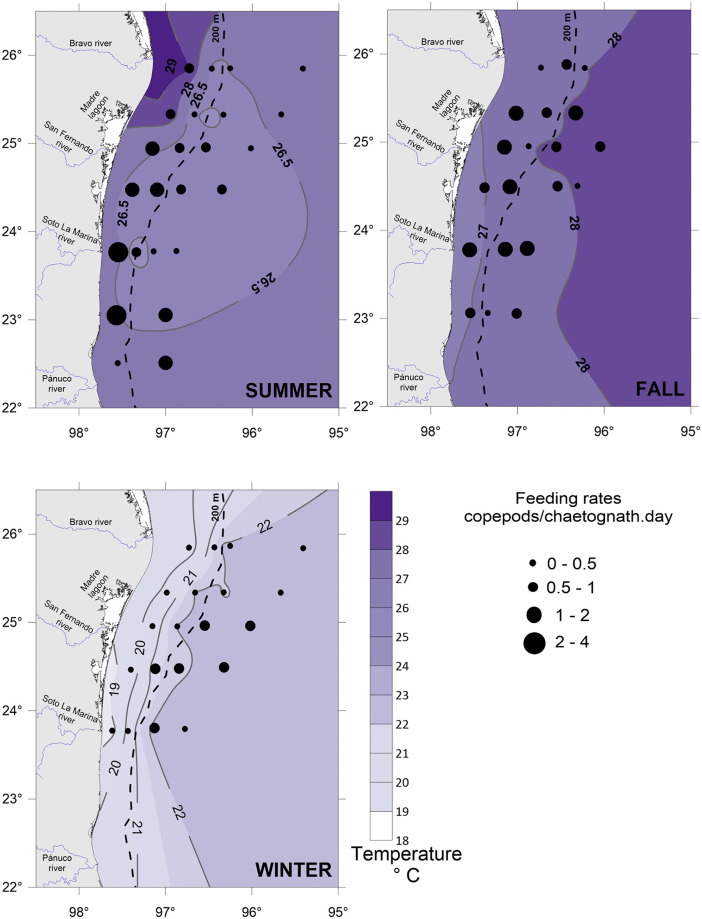
Feeding rates of the chaetognath *Flaccisagitta enflata* plotted over temperature conditions in the southwestern Gulf of Mexico.

**Fig 3:**
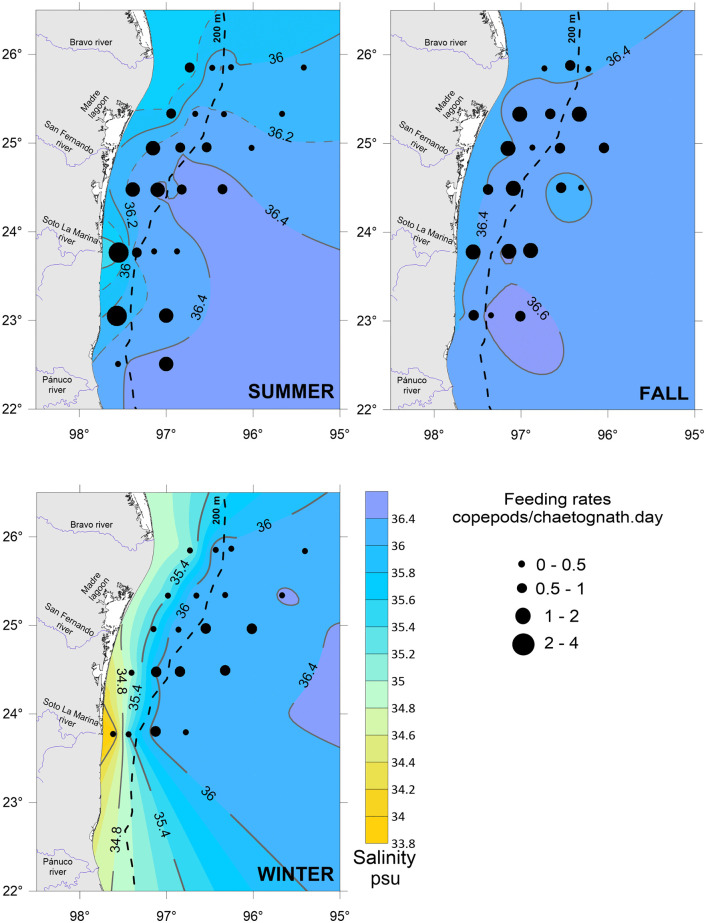
Feeding rates of the chaetognath *Flaccisagitta enflata* plotted over salinity conditions in the southwestern Gulf of Mexico.

In general, temperature registers in the study area were high during the summer (July) and fall (October-November), with temperature values exceeding 25°C in both neritic and oceanic zones (Fig 2). In winter (January), temperature values were lower in the neritic (18.7 to 22°C) than in the oceanic (21.7 to 23.1°C) zone (Fig 2). Salinity was higher in fall (> 36.3 psu) and lowest in winter, especially over the shelf (33.8–36.2 psu) (Fig 3). The chlorophyll concentration was generally low (<1.3 mg/m³) throughout the entire study area and across the three seasons. The highest values (1 to 1.3 mg/m^3^) were found in winter over the inner shelf, particularly around the mouths of the main rivers.

**Fig 4:**
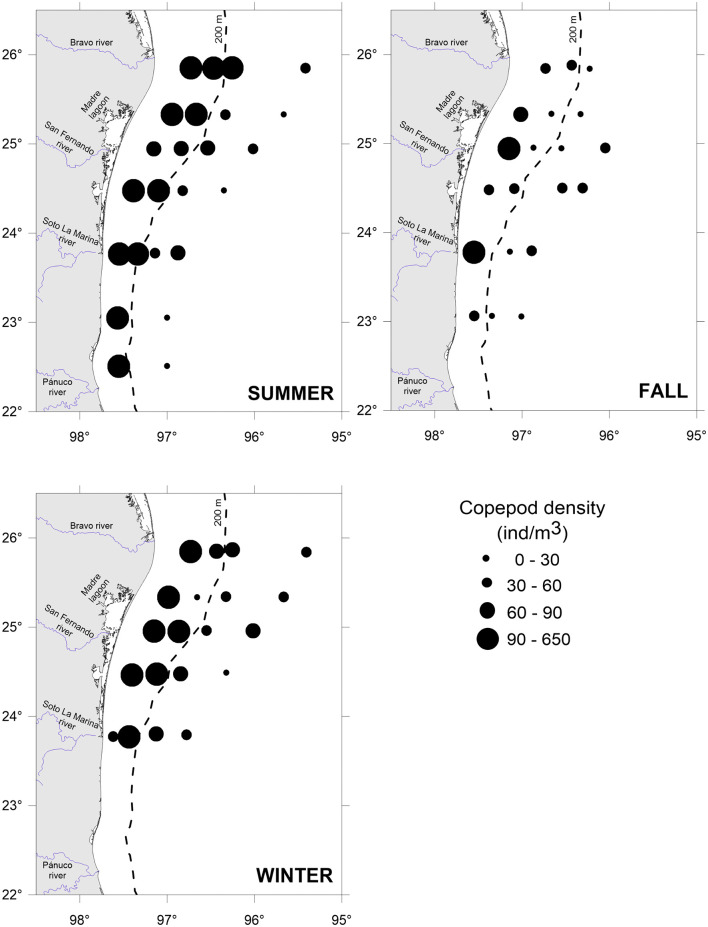
Copepod density in the southwestern Gulf of Mexico throughout three seasons.

**Fig 5:**
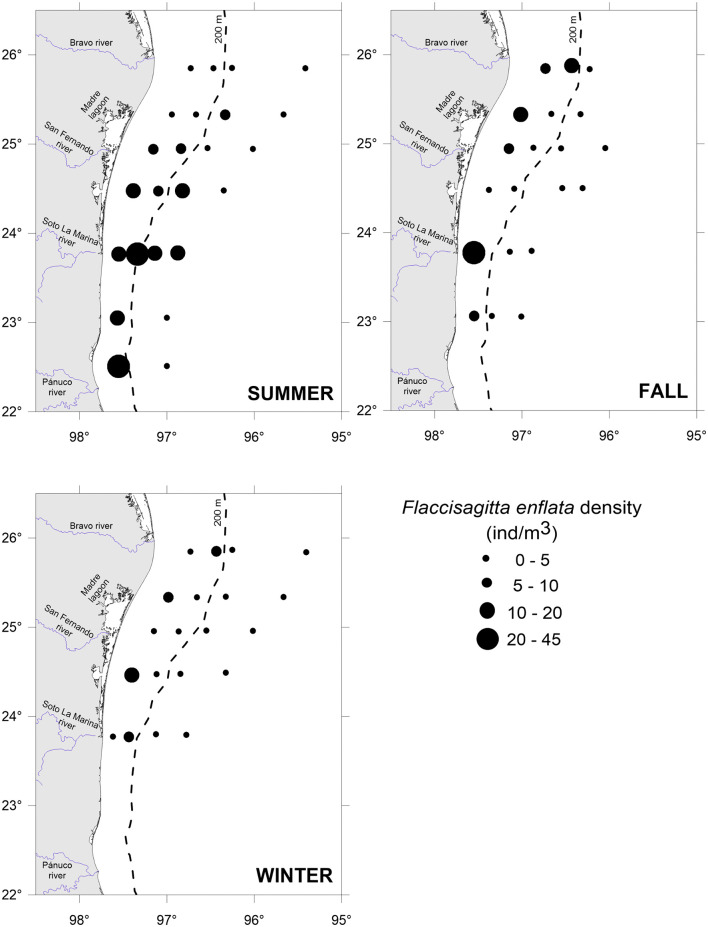
Density of the predator *Flaccisagitta enflata* in the southwestern Gulf of Mexico throughout three seasons.

Copepods, the prey, were widely distributed throughout the study area. The summer recorded the highest copepod densities, especially in the southern part, over the shelf (>250 ind/m^3^). The winter had intermediate density values, while the fall recorded the lowest densities (Fig 4). As well, the predator *F. enflata* was widely distributed in the study area and showed its highest densities during the summer and over the shelf (Fig 5).

### Encounter and feeding rates

**Fig 6:**
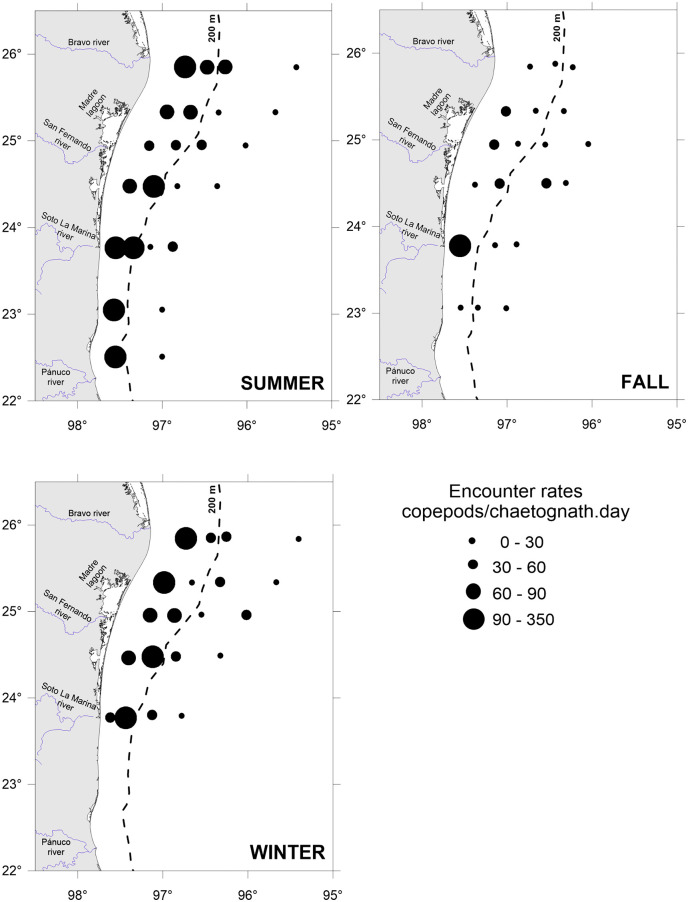
Encounter rates between the predatory chaetognath *Flaccisagitta enflata* and its prey, the copepods, at surface waters and turbulent conditions in the southwestern Gulf of Mexico.

**Fig 7:**
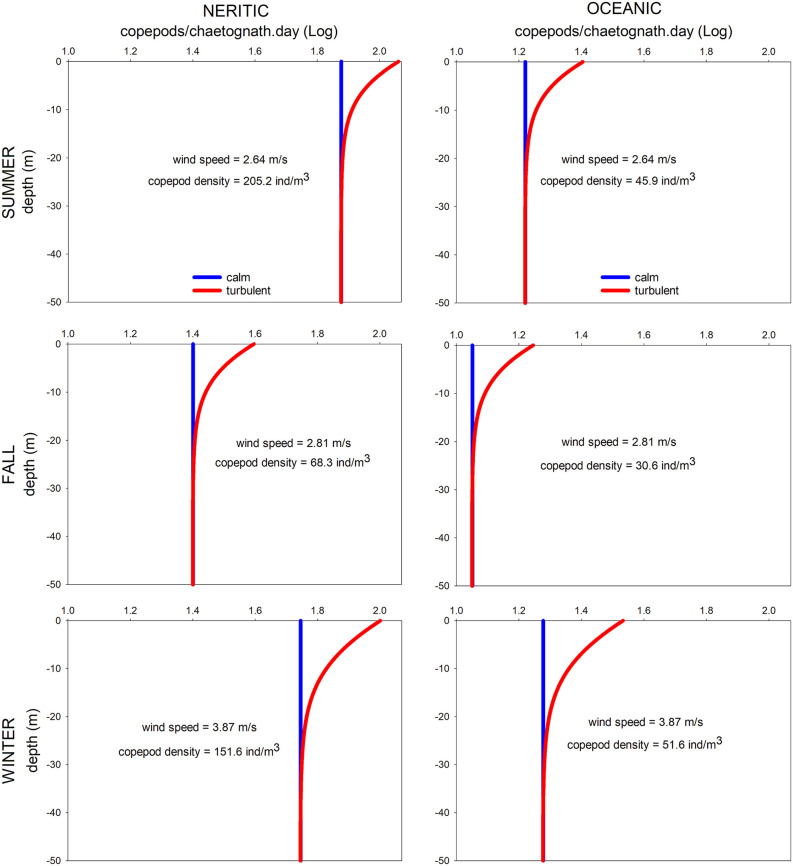
Vertical profiles of encounter rates between the predatory chaetognath *Flaccisagitta enflata* and its prey, the copepods, for calm and turbulent conditions and three seasons in the southwestern Gulf of Mexico. Horizontal scale in log10.

Seasonally, daily encounter rates between predators and prey under calm (GS model) and turbulent (RO model) conditions were higher in summer and winter, coincident with the major abundance of copepods (Figs 4 and 7). Spatially, the major encounter rates were found over the shelf (Figs 6 and 7). In the water column, encounter rate profiles showed that disparities between calm and turbulent conditions were more evident in the upper 28 m layer during the summer and fall, at wind speeds of 2.64 and 2.81 m/s, respectively. In winter, when the wind speed increases to 3.87 m/s, differences in vertical profiles between the two scenarios were palpable up to a depth of 40 m (Fig 7).

In the absence of turbulence induced by the wind, encounters are constant throughout the water column, with values ranging from 11 (oceanic zone in fall) to 75 (neritic zone in summer) copepods/chaetognath.day, depending on the season and zone (Fig 4). When the wind blows and promotes turbulent conditions, encounters increase by 1.5 to 1.8 times, resulting in encounters between 18 and 115 copepods/chaetognath.day at the surface (Fig 7).

After analyzing samples from the three sampling periods, results indicated that the number of copepods ingested by an individual of *F. enflata* (*NPC*) varied between 0 and 0.17, with the major variability in summer; the digestion time (*DT*) ranged from 0.86 to 2.09 hours, with the highest value in winter, where low temperatures were recorded (Fig 2); then, the mean feeding rates (*FR*) of *F. enflata* upon copepods were lower in this season (Table 1).

**Table 1:** Ecological parameters involved in the estimation of the feeding rate of *Flaccisagitta enflata.*

	Summer			Fall			Winter		
	Min	Max	$\overset{―}{X}\pm SD$	Min	Max	$\overset{―}{X}\pm SD$	Min	Max	$\overset{―}{X}\pm SD$
*T*	24.38	29.12	26.36 ± 0.98	26.27	28.20	27.65 ± 0.46	18.72	23.11	21.5 ± 1.10
*NPC*	0	0.17	0.04 ± 0.03	0.01	0.09	0.04 ± 0.02	0	0.05	0.03 ± 0.01
*DT*	0.86	1.29	1.09 ± 0.09	0.93	1.09	0.97 ± 0.04	1.44	2.09	1.66 ± 0.17
*FR*	0	3.78	0.81 ± 0.74	0.14	1.95	0.92 ± 0.44	0	0.78	0.40 ± 0.19

*T*: Temperature,°C; *NPC*: number of copepods captured per chaetognath; *DT*: digestion time, hours; *FR*: feeding rates, copepods/chaetognath.day.

The PERMANOVA analysis performed on the Euclidean similarity matrix of feeding rates showed significant differences (Pseudo-*F* = 2.91, *p* < 0.05; Table 2) in the interaction between the two considered factors, season and zone (Table 2). This result indicated that the same zone (neritic or oceanic) behaves differently among seasons. Indeed, in summer and fall, the feeding rates were higher over the neritic zone, whereas in winter, the oceanic zone registered higher values (Table 3).

**Table 2:** Results of the PERMANOVA analysis applied to the response variable (feeding rate) considering the seasons (summer, fall, winter), the zone (neritic, oceanic), and their interaction as sources of variability.

Source	df	Sum of squares	Mean squares	Pseudo-*F*	*p* (perm)	Perm
Season	2	9.1387	4.5694	5.6551	0.0044	9952
Zone	1	1.7991	1.7991	2.2265	0.1412	9855
Season x Zone	2	4.7033	2.3516	2.9104	0.0483	9955
Residuals	58	46.864	0.80801			

**Table 3:** Average values of the main potential parameters influencing the feeding rates of the chaetognath *Flaccisagitta enflata* upon copepods. In bold, the variables with major influence (positive or negative) over the feeding rates.

	Neritic						Oceanic					
	*T*	*S*	*Cop*	*GS*	*RO*	*FR*	*T*	*S*	*Cop*	*GS*	*RO*	*FR*
Summer	**26.6**	36.1	205.2	75.4	**115.1**	1.16	26.4	36.3	45.3	16.6	25.4	0.57
Fall	**27.3**	36.4	68.6	25.2	**39.4**	0.94	27.9	36.5	30.6	11.2	17.6	0.77
Winter	20.5	**35.5**	151.6	55.7	100.3	0.30	22.2	36.2	51.6	19.0	34.1	0.47

*T*: temperature,°C; *S*: salinity, ups; *Cop*: copepod density, ind/m^3^; *GS*: predator/prey encounters under calm conditions at surface, copepods/chaetognath.day; *RO*: predator/prey encounters under turbulent conditions at surface, copepods/chaetognath.day; *FR*: feeding rates, copepods/chaetognath.day.

Finally, the BEST BIOENV test revealed that the variables with the most significant influence over the feeding rates of chaetognaths were temperature, salinity, and encounter rates (*rho* = 0.225, *p* < 0.05).

### Gut contents

The examination of gut contents of chaetognaths showed that copepods constituted their major prey, with percentages ranging from 61.9% (summer) to 89.3% (winter) throughout seasons. Although at low levels (< 5%), consumption of other chaetognaths across the three sampling periods indicated cannibalism by *F. enflata*; in summer, other crustacean prey was also an important part of the diet of this planktonic predator (Table 4).

**Table 4:** Percentage of gut contents in the diet of *Flaccisagitta enflata* across the seasons.

Type of prey	Summer	Fall	Winter
Identified copepods	21.4	56.1	35.7
Unidentified copepods	40.5	26.8	53.6
Non-copepod prey			
Amphipoda: *Tetrathyrus forcipatus*	2.4	—	—
Luciferidae: *Belzebub faxoni*	7.1	—	—
Luciferidae: *Lucifer typus*	2.4	—	—
Myscidacea	4.8	—	—
Zoea larvae	2.5	—	—
Chaetognatha	2.5	4.9	3.6
Non-identified digested prey	16.7	12.2	7.1

Regarding the main prey, the copepods, the results of the Kimmerer and Slaughter electivity index indicated no selection for any prey, as all confidence intervals included 0.5. We recorded a total of 11 different taxa (genera/species) consumed by *F. enflata* and found in the environment, from which the genus *Euaugaptilus* was found in all three sampling periods, and *Temora* in summer and fall (Table 5). The fall registered the highest variety (8 taxa) of copepod prey.

**Table 5:** Percentage of copepods found in the environment and as prey of chaetognaths, including the Kimmerer and Slaughter electivity index and the 95% confidence interval.

Taxon	Gut	Habitat	Index	95% confidence interval
Summer				
*Acartia danae*	11.1	5.0	0.704	0.231 to 0.956
*Candacia*	—	12.5	—	—
*Centropages furcatus*	—	7.5	—	—
*Centropages velificatus*	33.3	17.5	0.702	0.347 to 0.916
*Euaugaptilus*	11.1	7.5	0.607	0.176 to 0.932
*Farranula rostrata*	—	5.0	—	—
*Labidocera acutifrons*	—	2.5	—	—
*Lucicutia*	—	5.0	—	—
*Oithona plumifera*	—	2.5	—	—
*Onychocorycaeus catus*	—	2.5	—	—
*Pontellina plumata*	11.1	—	—	—
*Subeucalanus subtenuis*	—	17.5	—	—
*Temora stylifera*	—	5.0	—	—
*Temora turbinata*	33.3	10.0	0.818	0.448 to 0.957
Fall				
*Agetus limbatus*	—	2.5	—	—
*Calanopia americana*	4.3	—	—	—
*Calanus*	21.7	37.5	0.316	0.129 to 0.594
*Candacia pachydactyla*	4.3	2.5	0.639	0.146 to 0.946
*Corycaeus catus*	8.7	2.5	0.788	0.301 to 0.966
*Euaugaptilus*	8.7	10	0.462	0.144 to 0.821
*Farranula*	4.3	2.5	0.639	0.145 to 0.945
*Lucicutia flavicornis*	—	2.5	—	—
*Mecynocera clausi*	—	2.5	—	—
*Oithona plumifera*	—	2.5	—	—
*Oithona setigera*	—	2.5	—	—
*Oncaea mediterranea*	—	5.0	—	—
*Pontellopsis*	4.3	—	—	—
*Temora stylifera*	4.3	7.5	0.359	0.088 to 0.819
*Temora turbinata*	39.1	12.5	0.818	0.560 to 0.938
*Undinula vulgaris*	—	5.0	0.316	—
*Urocorycaeus furcifer*	—	2.5	—	—
Winter				
*Calanus*	—	7.5	—	—
*Calocalanus*	—	5.0	—	—
*Candacia bispinosa*	—	2.5	—	—
*Candacia simplex*	—	5.0	—	—
*Candacia varicans*	—	5.0	—	—
*Centropages velificatus*	—	5.0	—	—
*Euaugaptilus*	10.0	10.0	0.5	0.134 to 0.887
*Euchaeta*	10.0	12.5	0.438	0.111 to 0.858
*Euchirella*	—	5.0	—	—
*Farranula*	10.0	—	—	—
*Gaetanus kruppii*	—	2.5	—	—
*Heterorhabdus*	—	2.5	—	—
*Labidocera scotti*	—	2.5	—	—
*Lucicutia*	30.0	—	—	—
*Nannocalanus minor*	—	2.5	—	—
*Oithona*	10.0	15.0	0.386	0.096 to 0.830
*Oncaea*	20.0	—	—	—
*Pareucalanus sewelli*	—	2.5	—	—
*Pontellopsis*	10.0	—	—	—
*Rhincalanus nasutus*	—	2.5	—	—
*Subeucalanus pileatus*	—	2.5	—	—
*Subeucalanus subtenuis*	—	7.5	—	—
*Temora turbinata*	—	2.5	—	—

**Fig 8:**
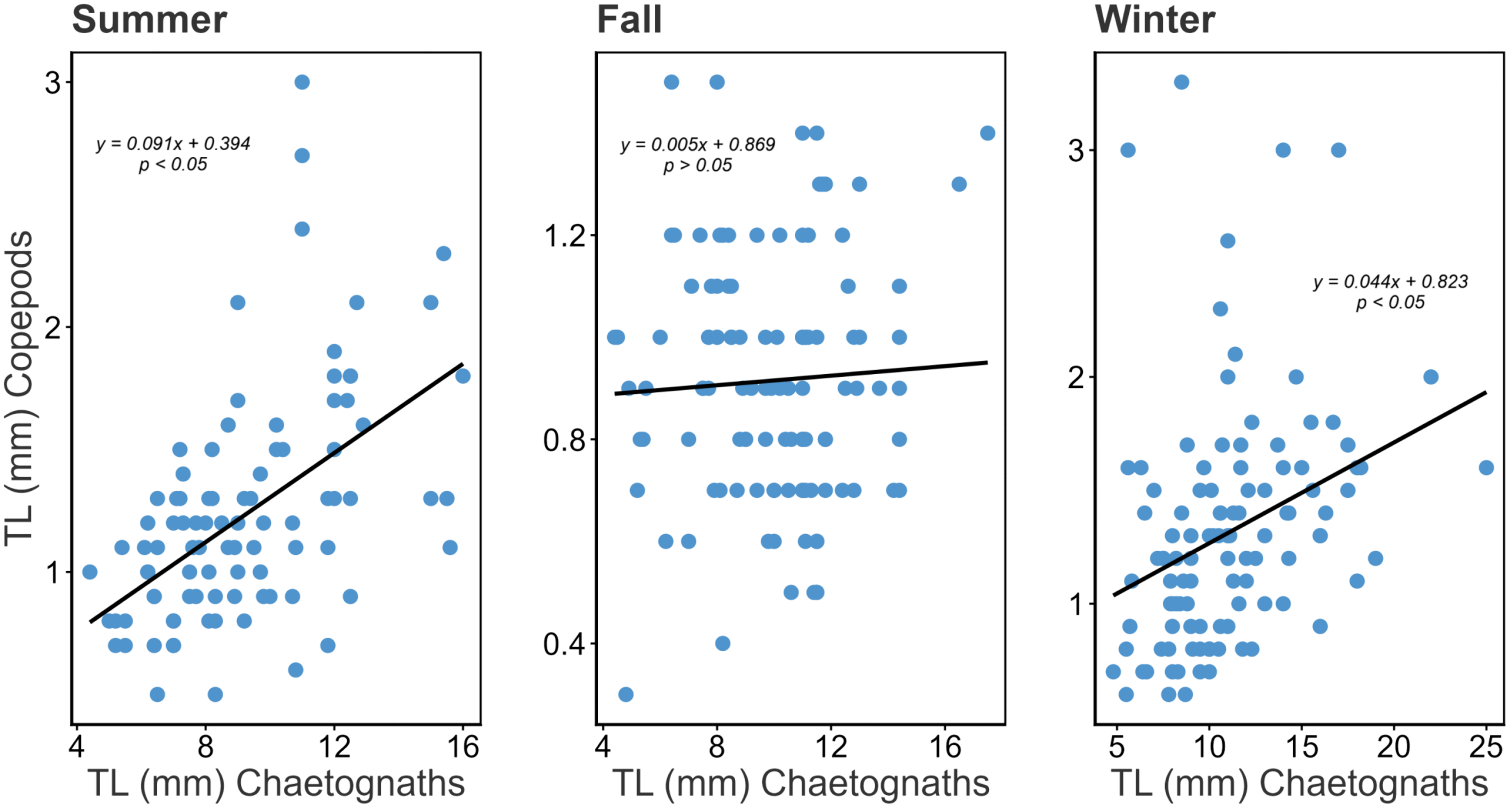
Relationship between the sizes of *Flaccisagitta enflata* (predator) and copepods (prey) throughout three seasons in the southwestern Gulf of Mexico.

The relationship patterns between prey and predator sizes were not consistent across seasons. While a positive trend was observed in summer and winter (*t*-test, *p* < 0.05), no significant relationship was found in fall (Fig 8).

## Discussion

### Encounter rates

In the vast pelagic environment, encounters among organisms play a fundamental role in many ecological processes, including trophic interactions [7,55]. Regarding the most abundant zooplankton organisms, the predatory chaetognaths and their copepod prey, feeding depends on the chaetognaths’ capacity to encounter prey, while the survival of copepods relies on their ability to avoid encounters. Encounters constitute an essential step in the predation cycle; indeed, before a predatory chaetognath could successfully chase and consume a prey, it must first be in contact with the prey. A general conceptual model of the predation cycle comprises five consecutive steps, beginning with the search for prey, followed by encounters, detection, attack, and capture [56]. For ambush predators, like chaetognaths, “searching” means waiting motionless for the swimming prey, and the “encounters” occur when the prey arrives in the sphere where the chaetognath can detect and attack the prey [4,56]. For a closely related species of *F. enflata*, the attack distance is approximately 3 mm, as determined by vibrating probe experiments [48]. For this condition, and copepod densities ranging from 15.8 to 602.9 ind/m^3^, encounters in the study area between predators and prey were between 11 and 75 copepods/chaetognath.day for calm conditions, and between 18 and 115 copepods/chaetognath.day for turbulent conditions, at surface waters (Fig 7). Regarding the copepods, their prey, they possess highly efficient mechanisms to escape from predators: they have a torpedo body shape, antennules armed with sensorial structures to detect hydrodynamic disturbances, and a powerful escape response that is an order of magnitude higher than that of other small organisms [21]. The escape jumps can reach velocities as high as 100–250 mm/s, as revealed by high-speed videographic techniques [49]. Therefore, the encounter rates between chaetognaths and copepods observed here (Figs 6 and 7) are not random events; they are the result of complex tactics employed by predators to capture prey and evasion mechanisms used by prey to avoid predators.

Seasonally, the encounters between the predator *F. enflata* and its prey, the copepods, were higher during the summer and winter, regardless of whether conditions were calm or turbulent (Fig 7), and were correlated with higher copepod densities (Fig 4). In the southern and western Gulf of Mexico, the copepod community exhibits high variability among sites and times, but a distinct peak is evident in summer [57,58]; it is highly likely that the low copepod density observed in the fall is due to low primary productivity values [59]. Spatially, encounter values were higher over the shelf than in the oceanic zone for both scenarios (Fig 7); again, a higher copepod density in neritic waters is the primary cause. When the wind blows, encounter values increase by 1.5 to 1.8 times at surface waters. For similar copepod densities, such as those registered in oceanic waters during summer and winter, the encounters were higher in winter (wind speed of 3.87 m/s), evidencing the role of wind-induced turbulence in influencing predator-prey encounters. However, the highest encounter values (Figs 6 and 7) occurred in summer in shelf waters, primarily due to the highest copepod density (Fig 4), despite the lowest wind velocity (2.64 m/s) [47]. In a previous study addressing encounter rates between chaetognaths and their prey, Saito and Kiørboe [60] modeled the encounters as a function of the search volume rate, rather than as counts per unit time; therefore, comparisons are not possible. For other ambush predators, such as siphonophores, estimates of encounters with fish larvae in the southern Gulf of Mexico yielded values ranging from 27 to 49 prey/predator.day [10], slightly lower than those here estimated because of the lower abundance of fish larvae and different wind conditions.

### Feeding rates

In the study area, multivariate analyses revealed that the feeding rates had a differential pattern among seasons (higher values over the shelf in summer and fall, and an opposite trend in winter) and that the temperature, encounter rates, and salinity had the major influence over the feeding rates. The feeding rate values registered here (Table 1) fall within the range reported for other seas around the world [43,61–63]. Still, the role of environmental conditions in the feeding patterns was not always evident. A coastal-ocean analysis in the western Mediterranean did not reveal a clear trend relating feeding rates to mesoscale hydrographic features [61]. In the Humboldt Current System, a negative correlation between the oxygen concentration and the feeding rate was found [62]. In the Kuroshio region, an examination of feeding activity by predator size classes showed that the higher the chaetognath body length, the higher the feeding rates [24]. Seasonally, in this study, the highest feeding rate values were estimated during the summer and fall, when the highest temperatures were recorded (Fig 2). Water temperature is a crucial factor in the feeding rates of chaetognaths, as it determines the digestion time of prey within their digestive tracts [27]. The general trend is that higher temperatures induce faster digestion, while lower temperatures result in greater digestion times. However, quite intrapopulation size-dependent variability has been observed [27,64,65]. Additionally, Feigenbaum and Maris [27] argued that the intrinsic physiological features of populations may determine differences in digestion times. In our study, shorter digestion times were observed in the summer and fall, coinciding with the highest feeding rates (Fig 2; Table 3). Estimations of feeding rates in two areas of contrasting temperatures showed higher values (1.32 to 4.68 prey/chaetognath.day) at temperatures of 26–30°C, and lower (0.13 to 0.58 prey/chaetognath.day) at temperatures of 14–21°C [61,63], evidencing the potential effect of temperature. However, other factors should be considered, as we show below.

In addition to temperature, the encounters of chaetognaths with their prey partially impact their feeding rates. In turn, encounters are proportional to the local prey concentrations and the level of turbulence [18,19]. In our study, the BEST BIOENV statistical analysis only considered the encounters due to the strong relationship with copepod density (Figs 4 and 6). It is generally accepted that the distribution of chaetognaths is closely related to the availability of food items [66]. Our results showed that higher encounter rates generally corresponded to higher feeding rates across seasons and zones, except in winter, when the highest feeding rates were recorded in the oceanic zone despite the lowest predator–prey encounter rates (Figs 2 and 6; Table 3). After the encounters, the ingestion of prey depends on the success of the attack and the capture of prey, which in turn are influenced by the size, swimming behavior, satiation level, and escape responses of prey [27,67–70]. Satiation adds a layer of complexity to understanding the interaction between chaetognaths and their prey. Although this phenomenon has not been well explored, previous studies [27,65] suggested that chaetognaths may not exhibit a direct relationship between prey abundance and feeding rates, as they may reach a satiation point. In the vertical dimension, the ‘hungry–satiation hypothesis’ [71] posits that hunger drives individuals to ascend for feeding, and once satiated, they descend. This results in a temporal sequence in the vertical distribution of individuals: as a chaetognath population ascends toward its preferred feeding depth, the leading individuals begin feeding first, and upon satiation, they descend back to deeper layers. A recent work in the southern Gulf of Mexico [35] registered the major densities of both chaetognaths and copepods over the neritic zone; however, the chaetognaths’ feeding rates were almost homogeneous in both neritic and oceanic waters, suggesting, perhaps, that chaetognaths attain satiety. This singularity can decouple chaetognath feeding rates from prey encounter rates due to several physiological and behavioral constraints on prey consumption. While encounters are determined by prey density, turbulence level, and the predator’s searching effort [4,18,46], feeding rates may be limited by gut fullness, digestion time, and post-feeding behavior [60,64,65,71,72]. These features of chaetognath biology prompt further reflection on satiation. Chaetognaths typically contain only one prey item in their guts, suggesting that digestion must occur before another prey can be ingested. The time required for digestion may also influence how long the predator remains satiated. According to the ‘hungry–satiation hypothesis’, once chaetognaths have eaten, they begin to descend, likely to avoid predators in surface waters. Further investigation of these aspects may shed new light on the mechanisms that decouple prey encounters from feeding rates.

In contrast to the summer and fall, winter observed the lowest feeding rates over the shelf, associated with low salinity values (Fig 3). While salinity is a crucial factor in coastal ecosystems, its impact on feeding rates is often less important than the features associated with low salinity conditions, such as increasing turbidity [73]. Lower salinity waters usually correlate with freshwater inflows carrying suspended sediments, which leads to higher turbidity. In the Gulf of Mexico, the Mississippi and Atchafalaya rivers discharge the largest amount of freshwater, and their influence can extend to the Mexican shelf during the winter months [74,75], as registered in the study area. Suspended sediments can disrupt several vital functions in aquatic organisms by reducing photosynthetic activity and interfering with respiration, feeding, and reproduction [76,77]. During feeding, turbidity may seriously affect visual predators by reducing their ability to detect prey, or it may clog the filtering structures of filter-feeding organisms [78,79]. However, in non-visual ambush predators, the role of suspended sediments remains largely unexplored, representing a significant gap in ecological research. In organisms that rely on mechanosensory abilities, such as chaetognaths, signal reception may be altered because turbidity modifies microscale water motion through changes in fluid density, viscosity, or turbulence [80]. These modifications can interfere with the predatory mechanisms, particularly their ability to sense prey through the detection of hydromechanical disturbances. This interference can reduce their feeding success; however, further research is needed to clarify the specific effects of turbidity on the predatory behavior of chaetognaths.

### Gut contents

As observed in previous studies [24,61–64,81], the diet of the planktonic predator *F. enflata* was mainly composed of copepods (Table 4). Copepods are the primary prey of chaetognaths due to their high abundance, small size, and continuous availability as a food source. In turn, the behavioral and morphological traits of chaetognaths —such as mechanoreceptors and grasping spines— enable them to efficiently detect and capture these prey [22,30]. Other microcrustaceans also constituted an important part of the diet of *F. enflata*, particularly in summer, likely due to their high abundance during this season [59]. Several studies have reported substantial overlap in the prey spectra of chaetognaths and other gelatinous zooplankton, suggesting potential competition for food among these groups. Both are major predators of copepods and other small zooplankton, occupying similar trophic niches within marine food webs [82,83]. However, the role of gelatinous organisms as competitors of chaetognaths remains poorly quantified. A study involving *F. enflata* and gelatinous zooplankton in a marine food web found that competition for prey between these groups was minimal or nonexistent, based on three key observations: (1) the feeding activity of chaetognaths did not increase with prey abundance, (2) no habitat partitioning between chaetognaths and gelatinous predators was observed, and (3) both predator groups had alternative prey options, reducing their dependence on the same food sources [35]. These results were likely due to the high food availability for both predator types at the study site. Quantifying the degree of competition in areas with lower prey availability would provide valuable insights. Cannibalism was also recorded in this study (Table 5). The gut contents of *F. enflata* revealed the presence of other chaetognaths, with percentages slightly differing from those reported in previous studies [24,64,84]. In some regions, cannibalism may significantly reduce populations of smaller or juvenile chaetognaths [63].

The specific identification of copepod prey in the gut of *F. enflata* showed no selectivity toward any prey species (Table 6). However, these results may not be conclusive, as a larger sample size would provide more reliable estimates [54]. A consistent finding across two or three seasons was the presence of the genera *Temora* and *Euaugaptilus* in the gut contents of the chaetognath. Members of the genus *Temora*, particularly *T. longicornis* and *T. turbinata*, are commonly reported as important prey items for chaetognaths in several regions of the world [85–87]. To our knowledge, the genus *Euaugaptilus* has not previously been reported as an important component of chaetognath diets; however, its consistent occurrence across the three seasons suggests a relevant role in the diet composition of *F. enflata* in the study area. Other copepod genera frequently preyed upon by chaetognaths, such as *Acartia*, *Calanus*, *Oncaea*, *Corycaeus*, and *Oithona* [24,27,86,87], were also recorded in this study.

According to the optimal foraging theory [88], larger chaetognaths would be expected to feed primarily on larger copepods, as the energy or nutritional gain outweighs the energy expended in searching for, capturing, and handling prey. However, in natural environments, this relationship is often more complex, as it can be influenced by several environmental factors. While some studies have reported positive predator–prey size relationships [24,61,81], others have found no consistent pattern [61,89]. Similar to our findings (Fig 8), in the western Mediterranean, prey size was not always related to predator size [61]. In our study, during fall —when no significant relationship was observed— we recorded a greater number of copepod species consumed (eight) than in summer and winter (Table 5). The high interspecific variability in the behavior of potential prey of similar size, particularly in motility and escape responses, may influence the attack and capture success of chaetognaths, ultimately resulting in the absence of a clear predator–prey size relationship [61]. In addition, differences in digestion times among copepod species [72] may also contribute to the variability observed in this relationship. These findings highlight the need for further field and experimental studies to better understand these patterns.

## Conclusion

This study examined the variability in encounters and feeding rates of the chaetognath *Flaccisagitta enflata* on copepods across three seasons (summer, fall, and winter) and two zones (neritic and oceanic) in the southwestern Gulf of Mexico. The feeding rates of *F. enflata* showed distinct seasonal patterns: the highest values were recorded over the shelf during summer and fall, whereas the lowest rates occurred in winter. The factors driving these seasonal differences included temperature, encounter rates, and salinity. Higher summer and fall temperatures promoted shorter digestion times and, consequently, faster feeding rates. The encounters, strongly related to food availability, were higher in neritic waters throughout the seasons, but greater in summer and fall, when the highest feeding rates were recorded over the shelf. Low salinity values recorded in winter in the neritic zone were associated with freshwater discharges, causing high turbidity, which in turn may interfere with the predation behavior of chaetognaths, resulting in low feeding rates. Copepods of the genera *Temora* and *Euaugaptilus* were among the main prey of *F. enflata*. Their presence in the gut contents during two or three seasons confirms a consistent trophic interaction between these planktonic groups. Examining the variability in the predator-prey interaction between chaetognaths and copepods is essential for a deeper understanding of the function of pelagic food webs.
